# Decreased heritability and emergence of novel genetic effects on pulse wave velocity from youth to young adulthood

**DOI:** 10.1038/s41598-021-88490-3

**Published:** 2021-04-26

**Authors:** Yisong Huang, Shaoyong Su, Harold Snieder, Frank Treiber, Gaston Kapuku, Xiaoling Wang

**Affiliations:** 1 Department of Medicine, Georgia Prevention Institution (GPI), Medical College of Georgia, Augusta University, Building HS-1640, Augusta, GA 30912 USA; 2 Department of Epidemiology, University Medical Center Groningen, University of Groningen, Groningen, The Netherlands; 3 College of Nursing, Medical University of South Carolina, Charleston, USA

**Keywords:** Genetics, Biomarkers, Cardiology, Risk factors

## Abstract

Increased arterial stiffness measured by pulse wave velocity (PWV) is an important parameter in the assessment of cardiovascular risk. Our previous longitudinal study has demonstrated that carotid-distal PWV showed reasonable stability throughout youth and young adulthood. This stability might be driven by genetic factors that are expressed consistently over time. We aimed to illustrate the relative contributions of genetic and environmental factors to the stability of carotid-distal PWV from youth to young adulthood. We also examined potential ethnic differences. For this purpose, carotid-distal PWV was measured twice in 497 European American (EA) and African American (AA) twins, with an average interval time of 3 years. Twin modelling on PWV showed that heritability decreased over time (62–35%), with the nonshared environmental influences becoming larger. There was no correlation between the nonshared environmental factors on PWV measured at visit 1 and visit 2, with the phenotypic tracking correlation (r = 0.32) completely explained by shared genetic factors over time. Novel genetic influences were identified accounting for a significant part of the variance (19%) at the second measurement occasion. There was no evidence for ethnic differences. In summary, novel genetic effects appear during development into young adulthood and account for a considerable part of the variation in PWV. Environmental influences become larger with age for PWV.

## Introduction

Increased arterial stiffness measured by pulse wave velocity (PWV) has been shown to be an important parameter in the assessment of cardiovascular (CV) risk. Previous longitudinal studies have confirmed the value of PWV measurements involving elastic arteries such as carotid-femoral and carotid-distal PWV in predicting cardiovascular events in patients with hypertension^1^, diabetes^2^, or end-stage renal disease^3^ as well as in general populations^4,5^. Cross-sectional studies have shown that individual differences in PWV were influenced by demographic factors such as age, gender and ethnicity, as well as genetic factors. Family and twin studies have shown that up to 53% of the individual differences in PWV can be attributed to genetic factors^6–8^.

Previously, we demonstrated reasonable stability of carotid-femoral and carotid-distal PWVs from youth to young adulthood^9^. Part of this stability might be caused by genetic factors that are expressed steadily over time. There have been no studies reporting the relative contribution of genetic and environmental factors to PWV stability over time. Understanding the sources of PWV stability over time is of considerable interest for the design of studies aiming to identify its determinants at an early age. On the other hand, PWV is known to increase with age and has been used as an index of vascular aging^10^. The age-specific increase in PWV raises several interesting questions. First, do novel environmental and/or genetic influences on PWV become apparent during the course of development? Second, is the age-specific increase in PWV levels a heritable trait itself? That is, to what extent is the increase of PWV over age genetically determined. All these questions have not been addressed in previous studies. Built on the Georgia Cardiovascular Twin Cohort which has carotid-distal PWV measured twice in 497 European American (EA) and African American (AA) twins, with an intervening period of 3 years, we will address these questions using bivariate twin modeling. Furthermore, ethnicity and gender differences in PWV have been well demonstrated with AAs and males having higher PWV than EAs and females^11,12^. Our bi-ethnic cohort with both males and females will allow us to further test the ethnic and gender dependency of the relative contribution of genes and environment to the individual differences in PWV over time.

## Methods

### Subjects

The present study comprised subjects from the Georgia Cardiovascular Twin Study which was established in 1996. It included roughly equal numbers of EAs and AAs (> 500 twin pairs) with the purpose to evaluate the change in relative influence of genetic and environmental factors on the development of cardiovascular risk factors. All twin pairs were reared together and zygosity was determined using five standard microsatellite markers in DNA collected with buccal swabs^13^. Subjects were recruited from the southeastern United States and were overtly healthy and free of any acute or chronic illness based on parental report. Study design, selection criteria and the criteria to classify subjects as EAs or AAs have been described previously^8,13^.

For the current study, data were available from 289 EA twins (46 monozygotic [MZ] vs. 75 dizygotic [DZ] pairs and 47 singletons; 52.2% females; aged 17.4 ± 3.4 at baseline) and 208 AA twins (25 MZ pairs vs. 59 DZ pairs and 40 singletons; 58.7% females; age 17.1 ± 3.5 at baseline) who had carotid-distal PWV measured at two visits with an average intervening time period of 3.7 years. The Institutional Review Board at the Augusta University had given approval for this study. Written informed consent was obtained from all subjects (and parents if subjects were < 18 years).

### Measurements

Carotid-distal PWV was measured noninvasively with applanation tonometry (Millar Instruments) and the SphygmoCor CPV analysis software (SphygmoCor, AtCor Medical, Sydney, Australia). Pressure waves were recorded at the common carotid and dorsalis-pedis arteries for the carotid-distal PWV. The SphygmoCor system calculated PWV from measurements of pulse transit time and the distance traveled by the pulse between the two recording sites: PWV = Distance (meters)/Transit Time (seconds). The carotid-distal rather than carotid-femoral was used due to its ease of access. Carotid-distal PWV measures arterial stiffness in both elastic and muscular artery and studies from our own and others^9,14,15^ have demonstrated that it not only showed reasonable stability over time but also was comparable to carotid-femoral PWV regarding to its correlations with other CVD risk factors as well as its predictive value of CVD morbidity and mortality.

Systolic BP (SBP), diastolic BP (DBP) and mean arterial BP (MAP) (Dinamap 1864 SX; Criticon Incorporated, Tampa, FL) were taken at 11, 13 and 15 min, during a 15-min supine relaxation period. The average was used to represent resting SBP, DBP and MAP. Anthropometrics and body composition assessment were obtained during the examination. Height and weight were measured by standard methods using a wall-mounted stadiometer and a digital scale, respectively. Body mass index (BMI) was calculated as weight/height^2^ (kg/m^2^)^13^.

### Statistical analysis

**Figure 1:**
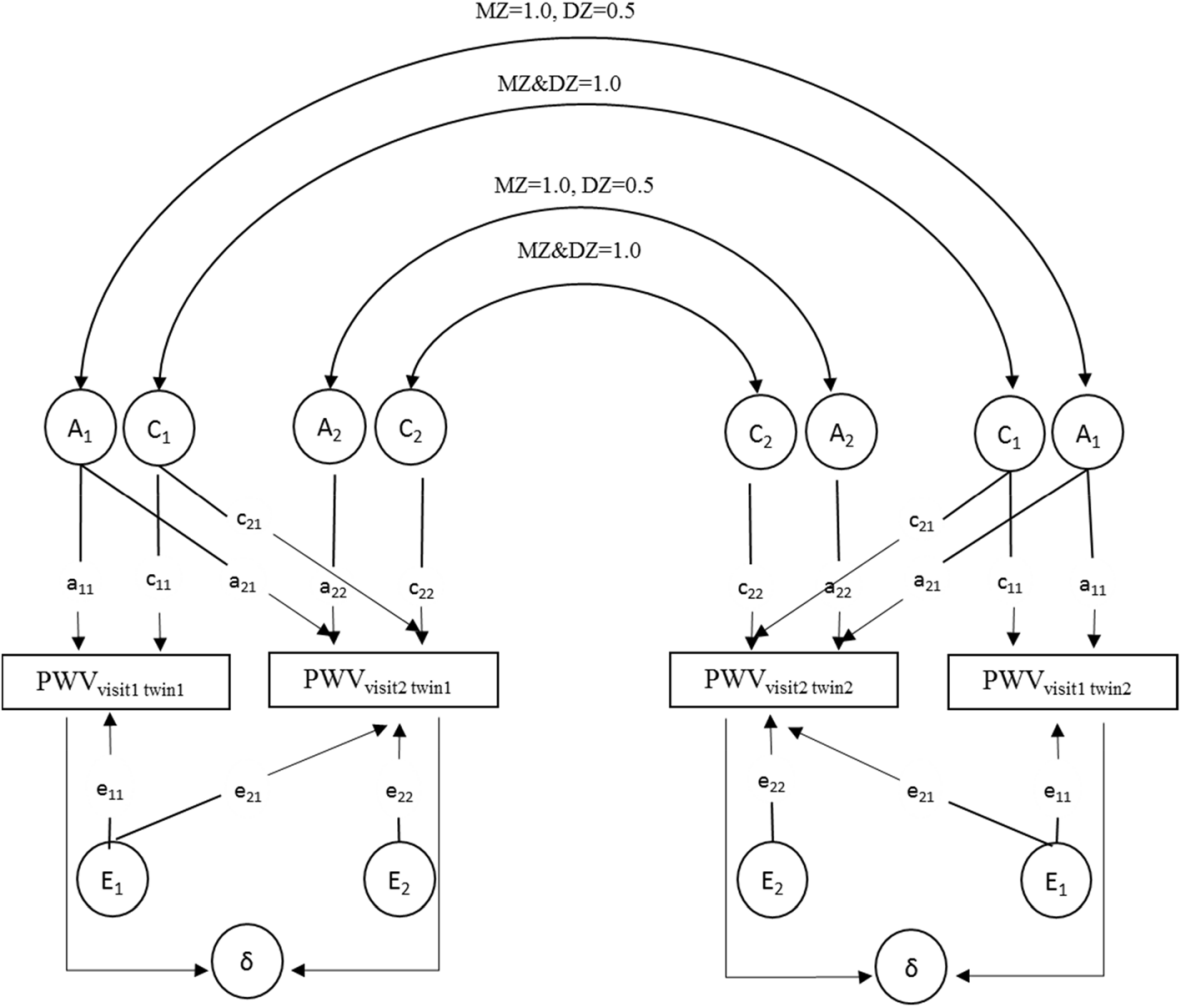
Bivariate structural equation model. The A_1_ and A_2_ are additive genetic factors. The E_1_ and E_2_ are unique environment factors. The C_1_ and C_2_ are shared environment factors. The paths of genetic factors are a_11_ through a_22_. The paths of unique environment factor are e_11_ through e_22_. The paths of shared environment factor are c_11_ through c_22_. Subtracting the level of measured PWV at visit 2 from visit 1 is established by setting the path coefficients of δ originating from visit 1 and visit 2 to − 1 to 1, respectively.

The aim of this study was to determine the relative contributions of genetic and environmental factors to the development of PWV from age 17 and 20 years and to what extent they depend on gender and ethnicity. Prior to all analyses, PWV was log transformed. Structural equation modeling (Fig. 1) was used to compare the variance–covariance matrices in MZ and DZ twins. The observed phenotypic variance of PWV at each visit was decomposed into additive genetic components (A), common environmental component shared by a twin pair (C), and unique environmental component specific to individuals (E). Dominance genetic effects (D) were not considered based on inspection of the twin correlations. The heritability (h^2^) was defined as the proportion of the total variance attributable to the additive genetic variation. A bivariate twin model was used for the current study (see Fig. 1). With the so-called Cholesky decomposition model, we can not only estimate the heritability of PWV at the first visit (h^2^_visit 1_ = a_11_^2^/(a_11_^2^ + c_11_^2^ + e_11_^2^)) and PWV at the second visit (h^2^_visit 2_ = (a_21_^2^ + a_22_^2^)/(a_21_^2^ + a_22_^2^ + c_21_^2^ + c_22_^2^ + e_21_^2^ + e_22_^2^)), but also can test whether the magnitude of the genetic influence differs between the 1st and 2nd visit (i.e. h^2^_visit 1_ = h^2^_visit 2_?). By constraining certain path coefficients to zero (Fig. 1) we can further test whether the genes influencing PWV at the second visit are the same (i.e., a_22_ = 0?), partly the same (i.e., a_21_ ≠ 0 and a_22_ ≠ 0?), or entirely different (i.e., a_21_ = 0?) from the genes influencing PWV at the first visit. If they were partly the same, this bivariate model allows further determination of the amount of overlap between genes influencing PWV measured at both visits by calculating the genetic correlation (i.e. genetic tracking coefficient) between the 2 PWV measurements. Shared and unique environmental correlations can be calculated in a similar fashion. Within the best fitting bivariate model of the PWV, we also calculated the heritability of PWV change between visit 1 and 2. The heritability of the difference score (i.e., PWV visit 2-PWV visit 1) can simply be derived from the parameter estimates of the phenotype levels in the bivariate model (Fig. 1). Significance of all of these models was tested by likelihood ratio tests. The model has been described in our previous study^16^.

The ethnic and gender differences were examined by comparing a full model in which the parameter estimates were allowed to differ among the four ethnic and gender groups (i.e. EA males, EA females, AA males and AA females), with a reduced model in which the parameter estimates were constrained to be equal across the groups. Significance of all of these models was tested by likelihood ratio tests.

Because BP is an important determinant of PWV and might explain part of its familial aggregation, we performed all model-fitting analyses before and after adjustment for MAP. All quantitative genetic modeling was carried out using OpenMx, version 1.2, an open source R-based package for analysis of twin data^17,18^. All parameter estimates in the twin analyses were obtained from full maximum-likelihood estimates in OpenMx, which enables handling of missing data and the inclusion of singletons.

## Results

The general characteristics of the participants by visit, ethnicity and gender were presented in Table 1. The mean age was 17 for the first visit and 20 for the second visit. Overall, PWV increased with age (*p* < 0.01) and AAs had higher PWV than EAs (*p* < 0.01). A significant 3 way interaction among ethnicity, gender and visit was observed. In the stratified analysis by ethnicity, EA males had higher PWV levels (*p* < 0.01) and faster increase over time (*p* = 0.02) than EA females, while no differences in PWV levels and rate of increase were observed between AA males and females.

**Table 1:** General characteristics of study participants by visit, ethnicity and gender.

N	European American				African American			
	Visit 1		Visit 2		Visit 1		Visit 2	
	Male	Female	Male	Female	Male	Female	Male	Female
	138	151	138	151	86	122	86	122
Age (years)	17.2 (3.6)	17.5 (3.1)	20.6 (4.2)	21.4 (4.0)	16.7 (3.1)	17.3 (3.8)	20.2 (4.3)	21.3 (4.4)
Height (cm)	171.1 (11.4)	161.3 (7.8)	175.6 (7.8)	162.4 (6.7)	172.0 (10.6)	162.6 (5.6)	175.9 (7.8)	163.5 (5.8)
Weight (kg)	69.0 (21.7)	59.7 (14.6)	77.90 (21.5)	64.2 (16.0)	73.0 (24.4)	68.8 (18.3)	81.5 (21.4)	76.7 (21.9)
BMI (kg/m^2^)	23.6 (5.9)	22.9 (4.8)	25.3 (6.5)	24.3 (5.6)	24.7 (6.0)	26.0 (6.5)	26.4 (6.1)	28.7 (7.8)
Waist (cm)	81.9 (15.11)	76.5 (12.3)	86.7 (15.6)	81.1 (14.0)	79.6 (14.9)	80.5 (13.2)	84.5 (15.4)	88.2 (18.3)
SBP (mmHg)	114.2 (10.6)	107.4 (8.3)	114.6 (10.8)	107.5 (9.6)	118.2 (10.6)	120.6 (10.9)	110.9 (10.6)	113.2 (11.9)
DBP (mmHg)	57.2 (6.9)	59.9 (6.8)	59.1 (7.7)	61.7 (6.6)	60.0 (6.7)	62.1 (8.2)	61.9 (7.5)	65.3 (8.7)
PWV (m/s)	7.08 (0.88)	6.99 (0.89)	7.60 (0.97)	7.24 (0.90)	6.98 (0.73)	7.21 (1.02)	7.73 (0.97)	7.82 (0.96)

Data were present as mean (SD).

All variables showed significant increase from visit 1 to visit 2 with *p* < 0.001.

In both ethic groups, twin correlations of MZ pairs were larger than those of DZ pairs, indicating genetic influences. Overall, the correlations of the second visit were smaller than the first visit for all groups (Table 2).

**Table 2:** Twin correlations of visit 1 and visit 2 by ethnicity and zygosity.

	European American		African American	
	Monozygotic	Dizygotic	Monozygotic	Dizygotic
N of pairs	46	75	25	59
Visit 1 PWV	0.66	0.54	0.70	0.54
Visit 2 PWV	0.41	0.27	0.64	0.37

**Figure 2:**
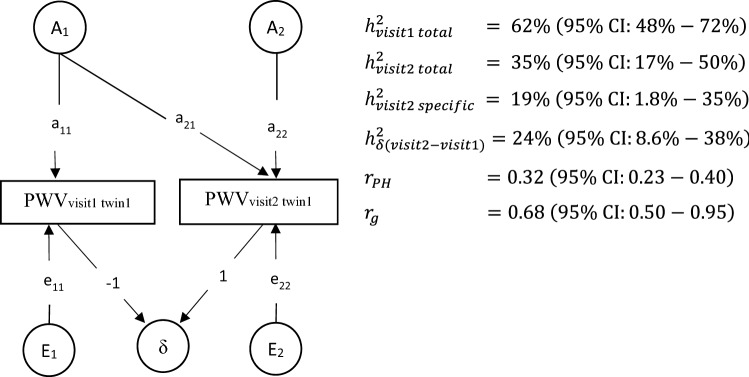
Path diagram for best fitting bivariate structural equation model (Cholesky model). The best fitting model was model composited of additive genetic factor (A), unique environment factor (E). The path of genetic factors between two visits is a_21_. The path of unique environmental factors between the phenotypes of the two visits is e_21_. The pathway a_21_ and a_22_ cannot be set to 0 while pathway e_21_ can be set to 0. r_PH_ is the phenotypic correlation for PWV between visit 1 and visit 2. r_g_ is the genetic correlation for PWV between visit 1 and visit 2. r_g_ = COV_A_(1st visit, 2nd visit)/√ (V_A_ 1st visit * V_A_ 2nd visit), with COV_A_ represents covariance and V_A_ represents variance.

Results from bivariate testing, using the model depicted in Fig. 1, are shown in Fig. 2. The overall best fit to the data for PWV at both visits is the model including an additive genetic and unique environmental component (ACE vs. AE $\chi_{3}^{2}$ = 1.29, *p* = 0.73, ACE vs. CE $\chi_{3}^{2}$ = 7.65, *p* = 0.05, AE vs. E $\chi_{3}^{2}$ = 51.43, *p* < 0.001). Furthermore, for both time points, no significant differences in the estimates of genetic and environmental variance components between males and females or between AAs and EAs were identified by the structural equation modelling, indicating the relative contribution of genetic and environmental factors to the variances of PWV over time does not depend on gender and ethnicity.

As shown in Fig. 2, significant heritability was found for PWV at both visits. However, there was a large decrease in heritability of PWV (from 0.62 to 0.35, *p* < 0.01) in visit 2. The path e_21_ can be set to zero (e_21_ ≠ 0 vs. e_21_ = 0, $\chi_{1}^{2}$ = 0.46, *p* = 0.50) while a_21_ cannot be set to zero (a_21_ ≠ 0 vs. a_21_ = 0, $\chi_{1}^{2}$ = 17.61, *p* < 0.001), indicating that the tracking of PWV over time (i.e. phenotypic correlation r_ph_ = 0.32) can be completely accounted by shared genetic factors overtime. Furthermore, a_22_ cannot be set to 0 (a_22_ ≠ 0 vs. a_22_ = 0, $\chi_{1}^{2}$ = 5.15, *p* = 0.02), indicating that there are new genetic influences emerging at the second visit. The specific heritability due to novel genetic effects emerging in visit 2 was 19% (95% CI: 1.8–25%). We also observed that the increase of PWV over time (i.e. delta in Fig. 1) was influenced by genetic factors with a heritability of 24% (95% CI: 8.6–37.9%).

The results from the twin modeling were identical after adjustment for MAP, indicating that the findings on PWV are independent of BP.

## Discussion

The important findings in this study are that independent of ethnicity and gender, the overall genetic influences on PWV decrease over time (from 62 to 35%), with emergence of novel genetic influences on PWV at visit 2. Furthermore, genetic factors are the major contributors to the tracking stability of PWV, with the phenotypic tracking coefficient completely explained by shared genetic factors over time, and the age specific increase in PWV is a heritable trait itself with a heritability of 24%.

It is well established that PWV increases with age^19,20^. Our study on PWV trajectories also documented an increase of PWV with age from youth to young adulthood in both AAs and EAs as well as males and females^21^. The increase in PWV with age suggests that different genetic or environmental mechanisms may have their influence on PWV in different periods of life. Without available genome-wide association study (GWAS) data on different age categories this question can only be answered through longitudinal twin or family studies in which the same subjects are measured repeatedly, although comparing cross-sectional family and twin studies conducted in different age groups may give us some clues. Our previous cross-sectional study on the Georgia Cardiovascular Twin Cohort observed a heritability of 53% for PWV at age 17.7 ± 3.3 years^8^. Tarnoki et al^22^ reported a heritability of 50% in healthy Hungarian and American Caucasian twins aged 43 ± 17 years, Medda et al^23^ reported a heritability of 49% in a middle age population (54.6 ± 12.4 years) from the Italian Twin Register, and the Twins UK study^6^ reported a heritability of 38% in female twins aged 58 ± 10 years. While a trend of small decrease in PWV heritability with aging might be detected, the large age range of these studies and the small differences in PWV heritabilities prevent us from drawing any solid conclusions. Recently, in a large family study in a Brazilian population, Alvim et al^24^ reported a heritability of 30 ~ 35% for PWV in participants with age ≤ 45 and 13 ~ 20% in participants with age > 45, providing the first piece of evidence on the decreased heritability of PWV with age. Our longitudinal twin study not only confirmed this finding, but also observed the emergency of new genetic influence on PWV with age. These results have important implications for gene-finding studies. In current gene-finding efforts for complex traits, large sample sizes are required to reach sufficient statistical power, especially when a GWAS design is used. Our study calls caution in pooling data from different age groups. Since the current study only targeted the PWV development from age 17 to 20, further follow-up of our twin sample will enable us to determine at what age the genetic component stabilizes (i.e. at what age no further novel genetic effects are expressed).

Progression of arterial stiffness measured by PWV changes over time has been used as an index of vascular aging^10^. Recently, the Twins UK study^25^ observed a heritability of 55% for the annual progression of PWV in female twins aged 57.9 ± 8.6 years with a follow-up of 5 years. In this study, we showed that even in youth and young adult, the PWV change over 3 years was a heritable trait with a heritability of 24%. So far, few studies have explored the physiological determinants of accelerated progression of aortic stiffness. The identified factors included high BP, smoking and male gender^19,25,26^. The identified heritability of PWV changes over time in youth and young adults implicates that genes also play an important role in the accelerated progression of aortic stiffness.

Ethnic differences in PWV have been reported with AAs having higher values than EAs^14,15,27^. We documented the same difference in the current study. However, the twin modelling analysis showed that the difference in mean values did not convert to many differences in genetic and environmental variability within each ethnic group. However, the fact that a similar amount of variation is explained by genetic factors within EAs and AAs does not exclude the possibility that the actual genes or their number responsible for these effects may differ between these two ethnic groups.

This study has several limitations. First, the generalizability of the results from the current study, which is comprised of youth and young adults, to other adult or elderly populations remains to be determined. Second, our overall sample size was substantial for longitudinal PWV measurements but may not have enough power to detect ethnic or gender differences in the relative contributions of genetic and environmental factors to the variations of PWV. Future twin studies with large sample sizes and long-term follow-up involving multiethnic groups are warranted.

In summary, our study demonstrated that the heritability of PWV decreased with age and different genes may have their influence on PWV in different periods of life. The increase of PWV with age is itself a heritable trait. These findings have important implications for gene finding studies.

### Ethics approval and consent to participate

This study was approved by the Institutional Review Board of Augusta University, and performed following the guidelines of the Declaration of Helsinki. Written informed consent was provided by all participants or by their parents if they were less than 18 years.

## References

[1] Laurent S (2001). Aortic stiffness is an independent predictor of all-cause and cardiovascular mortality in hypertensive patients. Hypertension.

[2] Cruickshank K (2002). Aortic pulse-wave velocity and its relationship to mortality in diabetes and glucose intolerance: an integrated index of vascular function?. Circulation.

[3] Blacher J (1999). Impact of aortic stiffness on survival in end-stage renal disease. Circulation.

[4] Schiffrin, E. L. Vascular stiffening and arterial compliance. Implications for systolic blood pressure. *American journal of hypertension* **17**, 39S-48S, 10.1016/j.amjhyper.2004.08.019 (2004). 10.1016/j.amjhyper.2004.08.019 15607434

[5] Mitchell GF (2010). Arterial stiffness and cardiovascular events: the Framingham Heart Study. Circulation.

[6] Cecelja M (2011). Arterial stiffening relates to arterial calcification but not to noncalcified atheroma in women. A twin study. J. Am. Coll. Cardiol..

[7] Tarnoki AD (2012). Heritability of central blood pressure and arterial stiffness: a twin study. J. Hypertens..

[8] Ge D (2007). Heritability of arterial stiffness in black and white American youth and young adults. Am. J. Hypertens..

[9] Ye C (2016). Pulse wave velocity in elastic and muscular arteries: tracking stability and association with anthropometric and hemodynamic measurements. Hypertens. Res..

[10] Mikael LR (2017). Vascular aging and arterial stiffness. Arq. Bras. Cardiol..

[11] Chen Y (2012). Age- and sex-related differences in vascular function and vascular response to mental stress. Longitudinal and cross-sectional studies in a cohort of healthy children and adolescents. Atherosclerosis.

[12] Snijder MB (2015). Ethnic differences in arterial stiffness the Helius study. Int. J. Cardiol..

[13] Ge D, Dong Y, Wang X, Treiber FA, Snieder H (2006). The Georgia Cardiovascular Twin Study: influence of genetic predisposition and chronic stress on risk for cardiovascular disease and type 2 diabetes. Twin Res. Human Genet..

[14] Townsend RR (2015). Recommendations for improving and standardizing vascular research on arterial stiffness: a scientific statement from the American Heart Association. Hypertension.

[15] Choo J (2014). Regional pulse wave velocities and their cardiovascular risk factors among healthy middle-aged men: a cross-sectional population-based study. BMC Cardiovasc. Disord..

[16] Kupper N, Ge D, Treiber FA, Snieder H (2006). Emergence of novel genetic effects on blood pressure and hemodynamics in adolescence: the Georgia Cardiovascular Twin Study. Hypertension.

[17] Neale, M. C. *et al.* OpenMx 2.0: Extended Structural Equation and Statistical Modeling. *Psychometrika* **81**, 535-549, 10.1007/s11336-014-9435-8 (2016). 10.1007/s11336-014-9435-8 PMC4516707 25622929

[18] Pritikin JN, Hunter MD, Boker S (2015). Modular open-source software for item factor analysis. Educ. Psychol. Measur..

[19] AlGhatrif M (2013). Longitudinal trajectories of arterial stiffness and the role of blood pressure: the Baltimore Longitudinal Study of Aging. Hypertension.

[20] Simonson E, Nakagawa K (1960). Effect of age on pulse wave velocity and "aortic ejection time" in healthy men and in men with coronary artery disease. Circulation.

[21] Liang X (2019). Determinants of pulse wave velocity trajectories from youth to young adulthood: the Georgia Stress and Heart Study. J. Hypertens..

[22] Tarnoki DL (2013). Genetic and environmental factors on the relation of lung function and arterial stiffness. Respir. Med..

[23] Medda E (2014). Heritability of arterial stiffness and carotid intima-media thickness: an Italian twin study. Nutr. Metab. Cardiovasc. Dis..

[24] Alvim RO (2017). Heritability of arterial stiffness in a Brazilian population: Baependi Heart Study. J. Hypertens..

[25] Cecelja M (2018). Arterial stiffening is a heritable trait associated with arterial dilation but not wall thickening: a longitudinal study in the twins UK cohort. Eur. Heart J..

[26] Ohyama Y (2016). Ten-year longitudinal change in aortic stiffness assessed by cardiac MRI in the second half of the human lifespan: the multi-ethnic study of atherosclerosis. Eur. Heart J. Cardiovasc. Imaging.

[27] Mokwatsi GG, Schutte AE, Kruger R (2017). Ethnic differences regarding arterial stiffness of 6–8-year-old black and white boys. J. Hypertens..

